# Genetic variants in *PARP1* (rs3219090) and *IRF4* (rs12203592) genes associated with melanoma susceptibility in a Spanish population

**DOI:** 10.1186/1471-2407-13-160

**Published:** 2013-03-27

**Authors:** Maria Peña-Chilet, Maite Blanquer-Maceiras, Maider Ibarrola-Villava, Conrado Martinez-Cadenas, Manuel Martin-Gonzalez, Cristina Gomez-Fernandez, Matias Mayor, Juan Antonio Aviles, Ana Lluch, Gloria Ribas

**Affiliations:** 1 Health Research Institute-INCLIVA, Valencia, Spain; 2 Department of Medicine, University of Castellon Jaume I, Castellon, Spain; 3 Servicio de Dermatología, Hospital Ramon y Cajal, Madrid, Spain; 4 Servicio de Dermatología, Hospital Universitario La Paz Hospital, Madrid, Spain; 5 Servicio de Dermatología, Hospital Gregorio Marañon, Madrid, Spain

**Keywords:** Melanoma, GWAs validation, Vitamin D, SNP, Genotyping, Case-control study

## Abstract

**Background:**

Few high penetrance genes are known in Malignant Melanoma (MM), however, the involvement of low-penetrance genes such as *MC1R*, *OCA2, ASIP*, *SLC45A2* and *TYR* has been observed. Lately, genome-wide association studies (GWAS) have been the ideal strategy to identify new common, low-penetrance susceptibility loci. In this case–control study, we try to validate in our population nine melanoma associated markers selected from published GWAS in melanoma predisposition.

**Methods:**

We genotyped the 9 markers corresponding to 8 genes (*PARP1, MX2, ATM, CCND1, NADSYN1, CASP8, IRF4* and *CYP2R1*) in 566 cases and 347 controls from a Spanish population using KASPar probes. Genotypes were analyzed by logistic regression and adjusted by phenotypic characteristics.

**Results:**

We confirm the protective role in MM of the rs3219090 located on the *PARP1* gene (p-value 0.027). Additionally, this SNP was also associated with eye color (p-value 0.002). A second polymorphism, rs12203592, located on the *IRF4* gene was associated with protection to develop MM for the dominant model (p-value 0.037). We have also observed an association of this SNP with both lentigines (p-value 0.014) and light eye color (p-value 3.76 × 10^-4^). Furthermore, we detected a novel association with rs1485993, located on the *CCND1* gene, and dark eye color (p-value 4.96 × 10^-4^). Finally, rs1801516, located on the *ATM* gene, showed a trend towards a protective role in MM similar to the one firstly described in a GWAS study.

**Conclusions:**

To our knowledge, this is the first time that these SNPs have been associated with MM in a Spanish population. We confirmed the proposed role of rs3219090, located on the *PARP1* gene, and rs12203592, located on the *IRF4* gene, as protective to MM along the same lines as have previous genome-wide associated works. Finally, we have seen associations between *IRF4, PARP1*, and *CCND1* and phenotypic characteristics, confirming previous results for the *IRF4* gene and presenting novel data for the last two, suggesting that pigmentation characteristics correlated with eye color are potential mediators between *PARP1* and MM protection.

## Background

Few malignant melanoma (MM) susceptibility genes have been described in the literature so far, with only two high-penetrance genes mutated in 20-40% of familial cases (Cyclin-dependent kinase inhibitor 2 (*CDKN2A*) and Cyclin-dependent kinase 4 (*CDK4)) *[1-3]. Seems likely that other genetic factors account for the remaining risk, and that environment should play a role in the etiology as well. It has been suggested that the remaining genetic risks may be due to low-penetrance susceptibility genes, such as the melanocortin-1 receptor (*MC1R*) gene. *MC1R* plays a role in pigmentation in several species; also, genetic variants are associated with pigmentary phenotypes in humans, including red hair, pale skin, freckling, and sun sensitivity [4,5]. Indeed, *MC1R* variants are associated with melanoma susceptibility in several study populations [6-19].

Subsequent studies examined other genes associated with MM. In population-based studies using candidate-gene approaches, a solute carrier 45A2 (*SLC45A2*) variant was associated with dark hair, dark skin, and protection from melanoma [20-23]. Variations in one of the genes causing oculo-albinism syndrome (*OCA2*) were associated with melanoma in other studies [24,25], Agouti signaling protein (*ASIP)* was found to modify melanoma risk in the presence of *MC1R* variants [12], and the Tyrosinase gene (*TYR*) has variants which code for skin color and are implicated in tanning response [26,27].

Recently,  new genome-wide  association  studies (GWAS) have been conducted and have identified novel genomic loci associated with melanoma [26,28,29]. GWAS are the ideal strategy to identify common, low-penetrance susceptibility loci without prior hypotheses about the role of the genes. Some of the associations detected were already known, such as *MC1R* with pigmentation and skin cancer, *ASIP, TYR, OCA2,* among others. Several novel chromosomal regions, however, have been revealed by using large cohorts of samples created by meta-analyses across studies, like 11q22.3 in Ataxia telangiectasia mutated gene (*ATM*), 21q22.3 located in Myxovirus resistance 2 gene (*MX2*) and 2q33.1 in Caspase 8 gene (*CASP8) *[26].

Taking into account that basal pigmentation and susceptibility to MM differ among populations would be important for determining the relevance of these new markers in more darkly pigmented populations such as the Spanish. Thus, in the present case–control study, we show the analysis of nine SNPs (corresponding to 8 genes: poly (ADP-ribose) polymerase 1 *(PARP1), ATM, CASP8, MX2,* Cyclin D1 *(CCND1),* cytochrome P450 family 2 subfamily R polypeptide 1 *(CYP2R1),* NAD synthetase 1 *(NADSYN1)* and interferon regulatory factor 4 *(IRF4)*. Six of them were detected by several GWAS studies looking for susceptibility to MM predisposition and an additional three were related to MM and serum levels of vitamin D levels which have been recently studied in relation to sun exposure and their protective role against cancer and other diseases [30,31].

## Methods

### Study subjects and data collection

A total of 566 non-related MM sample cases were recruited from 1st September 2004 up to the present at the departments of dermatology of three different Hospitals in Madrid: Gregorio Marañón University General Hospital, from La Paz University Hospital and Ramón y Cajal University Hospital. A total of 347 volunteer cancer-free control samples, were recruited at the National Research Cancer Center (CNIO) the Madrid College of Lawyers and Gregorio Marañón University General Hospital. All participants were Caucasians of Spanish origin, with the same ethnic background [32].

A standardized questionnaire was used to collect information on pigmentation characteristics such as eye, hair and skin color, number of *nevi*, presence of solar lentigines, sun exposure habits and presence of childhood sunburns. Sun exposure data consists of an estimation of the frequency (occasionally, always or never) of chronic sun exposure (due to daily work outdoors) and occasional sun exposure (corresponding to sporadic, weekend or holiday exposure), plus the use of sunscreen. Fitzpatrick’s classification of skin type, tumor localization, Breslow index (deep index) and personal or family history of cancer was extracted from the medical record of cases only. (Categorization of these variables as well as the distribution of the Spanish population sampled are shown in Additional file 1).

All study subjects gave informed consent, and the study was approved by the Ethics Committee of the Gregorio Marañón General University Hospital.

Genomic DNA from cases and controls was isolated from peripheral blood lymphocytes and diluted to a final solution of 50 ng/μl using the traditional saline method or the DNAzol procedure (Invitrogen, Eugene, OR, USA). DNA concentration was quantified in samples prior to genotyping by using Quant-iT PicoGreen dsDNA Reagent (Invitrogen, Eugene, OR, USA). Further concentration measures were obtained using a Nanodrop 2000 spectrophotometer. Genomic DNA was amplified using the GenomiPhi DNA Amplification Kit (GE Healthcare Bio-Sciences AB, Uppsala, Sweden).

### SNPs selection

Nine SNPs were selected from recent literature using high-throughput platforms in GWAS in order to validate the detected markers in a Spanish population (Table 1). Public databases were used to collect information about SNPs and genes: NCBI http://www.ncbi.nlm.nih.gov and Ensembl http://www.ensembl.org. Details such as MIM code, location, encoded protein, amino acid changes, nucleotide changes, minor allele frequency (MAF) from HapMap CEU databases and the context sequence are provided in Additional file 2.

**Table 1:** Previous published genome wide association studies considered in the present study

**A) Published studies of association with Melanoma**							
**Study**	**Gene**	**SNP**	**Allele**	**OR**	**p-value**	**MM association**	
MacGregor et al. 2011	*PARP1*	rs3219090	A	0.82	9.5 × 10^-7^	Protective	
Barrett et al. 2011	*ATM*	rs1801516	A	0.79	4.80 × 10^-7^	Protective	
	*CASP8*	rs13016963	A	1.18	5.68 × 10^-7^	Risk	
	*MX2*	rs45430	G	0.85	5.60 × 10^-7^	Protective	
	*CCND1*	rs1485993	A	1.19	4.15 × 10^-7^	Risk	
Duffy et al. 2010	*IRF4*	rs12203592	C	1.15	4 × 10^-3^	Protective	
**B) Previous studies of association with Vitamin D levels**							
				**Published Data**			
				**[VitD] < 75 nmol/L levels**		**[VitD] < 50 nmol/L levels**	
**Study**	**Gene**	**SNP**	**Allele**	**OR (CI 95%)**	**p-value**	**OR (CI 95%)**	**p-value**
Wang et al. 2011	*CYP2R1*	rs10741657	A	1.21 (1.45-1.29)	9.4 × 10^-11^	1.06 (1.00-1.13)	0.06
	*NADSYN1*	rs7944926	A	1.21 (1.14-1.29)	4.1 × 10^-10^	1.21 (1.14-1.29)	4.7 × 10^-9^
	*NADSYN1*	rs12785878	G	-	-	-	-

[VitD] means concentration of vitamin D on plasma.

OR: Odds Ratio.

The allele referes to the one considered as risk allele in the appropriated studies.

### Genotyping assays

Genotyping was carried out using KASPar technology (KBiosciences, Hoddesdon, UK). The PCR was performed in a total reaction volume of 4 μl containing about 10 ng of genomic DNA, with a final concentration of 4X New KASPar Reaction Mix, 12 μm of each Kaspar primer.

The PCR conditions depended on the requirements of each probe according to the manufacturer’s indications. The genotype of each sample was determined by measuring final allele-specific fluorescence in the ABI Prism 7900HT Detection System, using the SDS 2.3 software for allelic discrimination (Applied Biosystems, Foster City, USA).

As a quality control measure, we included one no template sample and one sample duplicate per 96-well plate (a total of four per 384-well plate used). Genotypes were provided automatically by the software and were confirmed manually by two different personnel in the laboratory.

### Statistical analyses

For all polymorphisms studied, Fisher’s exact test was used both to test for deviations from Hardy-Weinberg equilibrium (HWE) among controls, as well as to compare differences in the minor allele frequency (MAF) distributions between cases and controls. We set as risk factor the minor allele detailed in Table 2. We also performed a Cochran-Armitage trend test for allelic associations using the complement XLSTAT [33]. Preliminary analyses were performed using SPSSv19 (SPSS, Chicago, IL, USA). All p-values were two-sided, and those less than 0.05 were considered statistically significant. In order to assess associations among genotypes, haplotypes and MM risk, several analyses were performed. Genotype-related odds ratios (ORs), their corresponding 95% confidence intervals (CIs) and associated p-values were estimated via unconditional logistic regression. This was done for three penetrance models: genotypic, dominant (major homozygotes versus heterozygotes plus minor homozygotes) and recessive (major homozygotes plus heterozygotes versus minor homozygotes).

**Table 2:** Allelic frequencies in Spanish cases and controls

**Gene**	**SNP**	**Minor allele**	**p-HWE**	**MAF Controls**	**MAF Cases**	**p-value**	trend*
*PARPI*	rs3219090	A	0.27	0.329	0.275	**0.023**	**0.028**
*CASP8*	rs13016963	A	0.98	0.414	0.399	0.542	0.561
*MX2*	rs45430	G	0.93	0.380	0.392	0.624	0.603
*CYP2R1*	rs10741657	A	0.16	0.378	0.381	0.891	0.867
*CCND1*	rs1485993	T	0.48	0.448	0.438	0.697	0.652
*NADSYN1*	rs7944926	A	0.27	0.355	0.333	0.363	0.392
*NADSYN1*	rs12785878	G	0.75	0.369	0.330	0.102	0.109
*ATM*	rs1801516	A	0.04	0.145	0.121	0.185	0.208
*IRF4*	rs12203592	T	0.01	0.152	0.148	0.8	0.720

p-HWE: p-value obtained for deviation from Hardy-Weinberg Equilibrium. MAF: Minor Allele Frequency. Bold indicates statistically significant results.

*Trend p-values are obtained by performing a Cochran-Armitage test for trend.

Multivariate analysis was carried out combining all significant risk factors revealed in Additional file 1, in a multivariate logistic regression to estimate ORs, 95% CI and p-values. To assess the association of phenotypic characteristics with melanoma, the same logistic regression analyses were performed. To assess the mediation we performed logistic regression analyses using R (http://www.R-project.org) [34], based on the indications given at http://davidakenny.net/cm/mediate.htm. Known risk factors for MM (eye color, hair color, lentigines, and childhood sunburns) were evaluated for potential confounding effects by including them in multivariate analyses with each significant associated SNP.

### Functional analyses

We used the Haploview v4.2 tool from the HapMap webpage, in order to analyze linkage disequilibrium blocks for SNPs in the *PARP1* gene in order to know the frequencies of each haplotype in our population and HapMap populations from Northern Europe (CEU) and Tuscany Italians (TSI). We try to asses possible functional implications of all the polymorphisms in the genes of interest by using both the online software Pupasuitev3.1 (http://pupasuite.bioinfo.cipf.es) and the web tool “ECR Browser “(http://ecrbrowser.dcode.org/) to establish a comparison between the human genome and those of other animal species in order to analyze whether gene variations studied in this work are located in sequences important to the function of the protein and to search for the phylogenetically conserved regions of such genes as *PARP1, ATM* and *IRF4* genes*.*

## Results

### HWE and Allelic distributions

All polymorphisms were checked for HWE. Allele frequencies for each SNP and p-values for their comparison between 566 MM cases and 347 individual controls are detailed in Table 2 along with the p-values for the test of departure from Hardy-Weinberg equilibrium among controls. Only two SNPs gave slight departure from HWE rs1801516 in *ATM* (p-value 0.04) and rs12203592 in *IRF4* (p-value 0.01).

Based on unadjusted p-values, we observed evidence of differences in allele frequency for the SNP in the *PARP1* gene (rs3219090, p-value 0.023), implicated in DNA repair, Cochran-Armitage test support this association with a p-value of 0.028. We did not observe differences in the minor allele frequencies between cases and controls for any other SNP. Data are shown in Table 2.

### Association between Genotypes and Melanoma risk

Two SNPs were found to be associated with MM susceptibility: rs3219090*A, located on the *PARP1* gene and implicated in cell repair, is associated with protection from MM using the genotypic model with OR 0.79, 95% CI 0.63-0.97; p-value 0.027, and rs12203592*T, located in the *IRF4* gene, implicated in the immune response, which is associated with MM risk, when the recessive model is considered with OR 6.28, 95% CI 1.45-27.13; p-value 0.014. Due to the small number of minor allele homozygotes forming the risk group in the recessive model, we assumed this could be a spurious association. The same allele rs12203592*T also shows a trend towards protection when considering the dominant model with OR 0.83, 95% CI 0.61-1.12; p-value 0.2. The SNP rs12785878*G on the *NADSYN1* gene, associated previously with Vitamin D levels in plasma [31], is borderline associated with MM susceptibility when dominant model is taken into account, with OR 0.76, 95% CI 0.57-1.02; p-value 0.065.

Finally, allele rs1801516*A, located on the *ATM* gene, shows a trend towards protection in a similar manner to the published data in the GWAS previously reported [26] (Table 1). We were not, however, able to obtain statistically significant results (p-value 0.2). No other association remained statistically significant for any of the studied SNPs. Data is shown in Table 3 and in Table 4.

**Table 3:** Genotypic analyses of all the SNPs studied and their association with MM susceptibility in Spanish population

**Gene**	**SNP**	**Controls N (%)**			**Cases N (%)**			**Association**	
		**Major homozygotes**	**Heterozygotes**	**Minor homozygotes**	**Major homozygotes**	**Heterozygotes**	**Minor homozygotes**	**OR (CI 95%)**	**p-value**
*PARP1*	rs3219090	149 (46.4)	133 (41.4)	39 (12.1)	255 (53.2)	184 (38.4)	40 (8.3)	0.79 (0.63-0.97)	**0.027**
*ATM*	rs1801516	232 (74.6)	68 (21.0)	11 (3.5)	349 (77.7)	91 (20.3)	9 (2)	0.83 (0.62-1.11)	0.203
*CASP8*	rs13016963	112 (34.3)	158 (48.5)	56 (17.2)	174 (35.5)	241 (49.2)	75 (15.3)	0.94 (0.77-1.15)	0.539
*MX2*	rs45430	123 (38.3)	152 (47.3)	46 (14.3)	180 (37.6)	221 (46.2)	77 (16.1)	1.05 (0.86-1.29)	0.627
*CCND1*	rs1485993	93 (29.5)	162 (51.4)	60 (19.1)	143 (30.7)	237 (51)	85 (18.3)	0.96 (0.78-1.18)	0.691
*IRF4*	rs12203592	242 (70.1)	101 (29.3)	2 (0.6)	398 (74)	121 (22.5)	19 (3.5)	0.97 (0.74-1.26)	0.801
*CYP2R1*	rs10741657	123 (40.6)	131 (43.2)	49 (16.2)	172 (38.1)	214 (47.4)	65 (14.4)	1.01 (0.82-1.25)	0.893
*NADSYN1*	rs7944926	138 (43)	138 (43)	45 (14)	210 (45.5)	195 (42.3)	56 (12.1)	0.91 (0.74-1.12)	0.380
*NADSYN1*	rs12785878	130 (39.3)	157 (47.4)	44 (13.3)	211 (45.9)	194 (42.2)	55 (11.9)	0.84 (0.68-1.04)	0.106

Bold indicates statistically significant results.

N refers to the count of individuals, percentages among the total are indicated between parentheses.

OR (CI95%) means Odds Ratio and its 95% Confidence Interval.

P-values are calculated via unconditional logistic regression, taking into account the codominant model.

**Table 4:** MM associated SNPs with statistically significant associations in any of the three penetrance models analysed

**Gene**	**SNP**	**Penetrance model**		**Non adjusted**		**Adjusted**	
				**OR (CI 95%)**	**p-value**	**OR (CI 95%)**	**p-value**
*PARP1*	rs3219090	Codominant	GG/AG/AA	**0.79 (0.63-0.97)**	**0.027**	0.82 (0.62-1.07)	0.151
		Dominant	GG/AG + AA	0.76 (0.57-1.01)	0.059	0.80 (0.56-1.15)	0.228
		Recessive	GG + AG/AA	0.66 (0.41-1.05)	0.079	0.7 (0.38-1.28)	0.246
*IRF4*	rs12203592	Codominant	CC/CT/TT	0.97 (0.74-1.26)	0.801	0.76 (0.53-1.08)	0.122
		Dominant	CC/CT + TT	0.83 (0.61- 1.12)	0.214	**0.65 (0.44-0.98)**	**0.037**
		Recessive	CC + CT/TT	6.28 (1.45-27.13)	**0.014**	4.07 (0.83-20.05)	0.084
*NADSYN1*	rs12785878	Codominant	TT/GT/GG	0.84 (0.68-1.04)	0.106	0.83 (0.64-1.08)	0.165
		Dominant	TT/GT + GG	0.76 (0.57-1.02)	0.065	0.76 (0.53-1.10)	0.142
		Recessive	TT + GT/GG	0.89 (0.58-1.35)	0.575	0.84 (0.49-1.44)	0.522
*ATM*	rs1801516	Codominant	GG/AG/AA	0.83 (0.62-1.11)	0.203	0.88 (0.61-1.28)	0.504
		Dominant	GG/AG + AA	0.84 (0.6-1.18)	0.318	0.92 (0.59-1.41)	0.686
		Recessive	GG + AG/AA	0.56 (0.23-1.36)	0.198	0.53 (0.15-1.8)	0.310

Bold indicates statistically significant results.

OR (CI 95%) means Odds Ratio and its 95% Confidence Interval.

The results are obtained taking into account three penetrance models: Codominant (major homozygotes versus heterozygotes versus minor homozygotes), Dominant (major homozygotes versus heterozygotes plus minor homozygotes) and Recessive (major homozygotes plus heterozygotes versus minor homozygotes).

Adjusted for eye color, hair color, lentigines and childhood sunburn via multivariate analysis by performing logistic regression.

We performed a multivariate analysis, taking into account phenotypic risk factors such as eye and hair color, solar lentigines and the presence of childhood sunburn, together with candidate SNPs. We verified that hair color, lentigines and childhood sunburn were independently associated with MM. Our SNP most associated with MM protection, rs3219090, maintained the trend (OR 0.82, CI 95% 0.62-1.07; p-value 0.15). Since this SNP was associated with eye color, a risk factor for MM, we suspected that the association between rs3219090 and MM was actually mediated by the eye color. In order to test for the mediation effect of eye color, we performed a mediation analysis considering as covariates the SNP as well as the eye color. The association between this gene and melanoma was no longer significant after adjustment for eye color (OR 0.81, CI 95% 0.65-1.01, p-value 0.066). For the rs12203592 SNP located on the *IRF4* gene, we observed a statistically significant result (OR 0.65 CI 95% 0.44-0.98, p-value 0.037) for the dominant model, reinforcing the trend towards protection found in the non-adjusted analysis. The SNP rs12785878 on the *NADSYN1* gene maintained a trend to signification. See adjusted values on Table 4.

### Associations between genotypes and phenotypic characteristics

We assessed whether the SNPs selected from GWAs studies were associated with various phenotypic characteristics. To carry out this task we used the genotypic, dominant and recessive models for each SNP and their associations with all phenotypes.

We observed strong evidence of association with eye color for 3 SNPs. Two of them, rs3219090 on the *PARP1* gene with OR: 0.69 (CI 95%: 0.54-0.88, p-value 0.002), and rs1485993 on the *CCND1* gene with OR 0.561 (CI 95% 0.41-0.78, p-value 4.96 × 10^-4^), both correlated with dark eye color. The third, rs12203592 on the *IRF4* gene, with OR 1.83 (CI 95% 1.34-2.51, p-value 1.63 × 10^-4^) was associated with light eye color.

The rs12203592 SNP on the *IRF4* gene with OR 1.61 (CI 95% 1.16-2.24, p-value 0.005) is correlated with the presence of lentigines. We observed an association with absence of childhood sunburn with the SNP rs12785878 located on the *NADSYN1* gene with OR 0.69 (CI 95% 0.51-0.93, p-value 0.015).

We observed other less robust phenotype correlations for skin color and two SNPs; rs10741657 on the *CYP2R1* gene with OR 1.24 (CI 95% 1-1.54, p-value 0.045) and rs7944926 on the *NADSYN1* gene with OR 1.37 (CI 95% 1.03-1.84, p-value 0.033) were both associated with light skin color. Additionally, we observed two SNPs associated with the number of *nevi:* rs7944926 on the *NADSYN1* gene with an OR of 1.59 (CI 95% 1.01-2.48, p-value 0.044) and the rs1801516 on the *ATM* gene with an OR of 3.12 (CI 95% 1.06-9.21, p-value 0.039). All this data is shown in Additional file 3.

### Functional and haplotype analysis and association with melanoma risk

We have previous results for rs1136410 on the *PARP1* gene [35], and we have combined them with the current results for rs3219090 on the same gene. We performed haplotype analyses; both SNPs belong to a single block according to the Haploview v4.2 program (data used from HAPMAP_CEU and TSI subset of samples). Three haplotypes were obtained, with “TG” being the majority haplotype at 70% frequency. When we studied the case–control analysis, a trend towards protection for the haplotype “CA”, with the homozygotes minor alleles in both positions, is maintained (OR 0.79). Furthermore, we detected that two SNPs at approximately 8 kb in the 5’upstream region of the ATG, (rs224984 and rs139755) are in complete LD with the associated rs3219090. When we checked for transcription binding sites in the surrounding sequences of these two SNPs, we observed a likely probability for the presence of the regulatory gene functions of interest. Finally, the functional assessment for the 34 genetic variants in the whole sequence of the *PARP1* gene showed a single LD block, and 18 of the SNPs present might be located in phylogenetic conserved regions. Only the minor allele of a non-synonymous variant (rs1136410, pV762A) is carried in approximately half the haplotypes that carry our genotyped SNP. The results of these analyses indicate that SNP rs3219090 is located in a simple repeats area in intron 13, close to an exon. This region is conserved in the cow (*bos taurus*), macacus (*rhesus macaque*) and chimpanzee (*pan troglodytes*). It acts as an intronic enhancer and might function as a regulator of transcription factors.

The rs12203592 SNP on the *IRF4* gene is located in intron 4, and this region is conserved in the opossum (*monodelphis domestica*), rat (*rattus norvegicus*), mouse (*mus musculus*), dog (*canis familiaris*), cow (*bos taurus*), macacus (*rhesus macaque*) and chimpanzee (*pan troglodytes*). When studying the complete genomic region of the *IRF4* gene, we observed 25 SNPs of which 18 are located in conserved regions, including the genotyped rs12203592; however, only the rs1514346 SNP located in the putative promoter region are not in LD with the genotyped SNP in this study appears to affect the binding to the TFBS ETS1.

The rs1801516 SNP, located on the *ATM* gene, is located in exon 34 and may disrupt splicing regulation. This SNP is conserved in opossum (*monodelphis domestica*), rat (*rattus norvegicus*), mouse (*mus musculus*), dog (*canis familiaris*), cow (*bos taurus*), macacus (*rhesus macaque*) and chimpanzee (*pan troglodytes*). When we studied the whole genetic variability of the gene, we observed 12 out of 62 SNPs of a non-synonymous nature, among which was the genotyped rs1801516*A, responsible for the change at position 1853 of the protein that causes an alteration of the common amino acid Asp [D] (negative charge) to a polar residue, Asn [N]. Furthermore, another 35 SNPs seem to be located in conserved regions.

## Discussion

In this case–control study we have analyzed a group of nine SNPs selected from previous GWAS and literature related with MM and/or Vitamin D levels with the intention of validating the results in a Spanish population. These validation studies are important in order to confirm the role of these SNPs in populations with different levels of basic pigmentation and make them more relevant. The study in our population, one from the southern Mediterranean, allowed us to observe two strong associations. Despite being able to detect pigmentation and MM associations with some of the candidates, we could not validate them all, probably due to our modest sample size which may not be sufficiently large enough to detect associations from GWAS which use thousands of samples. It is worth noting that having data on sun exposure habits and phenotypic traits has allowed us to give robustness to our results. In addition, we have been able to find novel pigmentation associations and validate others previously described, thereby providing relevant complement information.

First of all, we would like to highlight the rs3219090 SNP, which is located in intron 13 on the *PARP1* gene. This gene codes for a chromatin-associated enzyme, poly-ADP ribosyltransferase, which is implicated in several important cell functions such as DNA repair. *PARP1* was studied previously in relation to melanoma [35,36]; however, the rs3219090 was firstly detected in a GWAS study [28], and the validation of its protective role to MM predisposition has been confirmed in this study’s southern Mediterranean (Spanish) population (p-value 0.027). Furthermore, we observed a novel association with eye color (p-value 0.002) not described elsewhere for rs3219090*A. The melanoma association does not remain significant after performing mediation test with eye color, suggesting that this phenotypic trait could mediate to melanoma susceptibility in this population. Nevertheless, our data maintained the same trend towards protection previously described in the GWAS [29]. Additional functional assessments performed in the current study have shown that the associated SNP could indeed be located in an important region since it is a conserved sequence in mammalian species. Furthermore, two SNPs in complete LD with rs3219090, and which are close to the starting codon, could disrupt the binding sites of several transcription factors. This gene has been related to other diseases like gastric cancer [37], cardiopathies [38], glioblastoma [39], bladder cancer [40] and breast cancer [41]. This association to MM could be very relevant. For example, in a highly drug resistant cancer such as melanoma, a candidate gene with known and available inhibitors could be used as an effective therapy, as is being studied in other cancers [42]. Moreover, mechanisms such as gene silencing can reduce the aggressiveness of MM, further suggesting that this gene may be a possible candidate for future therapy [43].

Second, we confirmed a protective association with rs12203592, located in intron 4 on the *IRF4* gene. This gene codes for a protein which belongs to the family of transcription factors. The IRFs are important in the regulation of interferon in response to viral infections, and in the regulation of interferon-inducible genes. This SNP was firstly detected in GWAs with an association for hair color and skin pigmentation [44], followed by an association with tanning phenotype [45]. In spite of its being related to pigmentation, the association of this rs12203592 with MM was not always detected [46]. In our study, rs12203592*T was associated with protection to melanoma (OR 0.65, p-value 0.037) when dominant penetrance model is taken into account. A recently published study suggests that minor allele of this SNP (rs12203592*T) is actually associated with risk of developing skin cancer, including MM [47], nevertheless our study validates the protective association obtained by Duffy et al., where rs12203592*C (major allele) was associated with the presence of *nevi* and a predisposition to melanoma [48]. Although we have not being able to find any association between the presence of nevi and rs12203592, we did detect an association with lentigines [48]. In addition, we obtained a strong correlation with light eye color and this SNP (p-value 1.63 *10^-4^). This result points in the same direction as previous works in which this SNP has been associated with human skin and eyes pigmentation and was selected as one of the six SNPs used in the IrisPlex (set of SNPs studied together in order to predict eye color) [49]. This SNP was slightly out of HWE (p-value 0.01); however, other SNPs located on pigmentation genes such as SLC45A2 alleles, have shown deviation from HWE in several populations of Caucasian origin. This may be explained by the effects of natural selection on skin color, assortative mating or admixture [21,50]. The region around this SNP is conserved in all mammalian species sequenced to date. There are known associations between the *IRF4* gene and several diseases, such as rhinitis [51] and chronic lymphocytic leukemia [52,53].

Similarly, we have detected for the first time an association with dark eye color and rs1485993*T located on the *CCND1* gene (p-value 4.96 × 10^-4^). Although six SNPs are used in a multiplex that identifies blue vs non-blue eyes, with high correlation and forensic applications, it is of interest to further analyze this proposed SNP due to the strong association found in our study.

Finally, we would like to highlight that the SNP (rs1801516) located on another gene with repair functions, *ATM*, shows an interesting trend towards a protective role in MM similar to the one described in the first GWAS study [26]. In addition, the SNP is in a region with high sequence conservation for all mammalian species available so far. Moreover, according to an *in silico* functional analysis performed, it may well be able to regulate several transcription factors. The protein encoded by this gene belongs to the PI3/PI4-kinase family. This protein is an important cell cycle checkpoint kinase, and the closely related kinase ATR are thought to be master controllers of the cell cycle checkpoint signaling pathways required for cell response to DNA damage and for genome stability. As well as being responsible for Ataxia telangiectasia, *ATM* is also involved in several diseases such as diabetes mellitus type 2 [54], leukemia [55], breast cancer [56] and cervix cancer [57], in which the rs1801516 SNP, has been associated with the risk to develop LSIL (Low grade Squamous Intraepithelial Lesion). Moreover, therapeutic advances have been described, based on ATM inhibitors, which are capable of inducing cell apoptosis in cancer cell killing in Melanoma cases [58]. The fact that *PARP1* and *ATM* genes are involved in cell repair, suggests that DNA repair signaling pathways are an important function in susceptibility to melanoma risk.

## Conclusions

In summary, we detected two associations with MM, rs3219090 on the *PARP1* gene (mediated by eye color), and rs12203592, on the *IRF4* gene (significant after adjusted by phenotypic traits), both conferring a protective role in MM. In addition, we detected three associations the pigmentary characteristics such as eye color, for *PARP1, IRF4* and *CCND1* genes in our study population.

## Abbreviations

MM: Malignant Melanoma; MC1R: Melanocortin 1- receptor; CDK4: Cyclin-dependent kinase 4; CDKN2A: Cyclin-dependent kinase inhibitor 2A; SLC45A2: Solute carrier 45A2; OCA2: Oculo-albinism syndrome; ASIP: Agouti Signaling Protein; TYR: Tyrosinase gene; PARP1: Poly (ADP-ribose) polymerase; ATM: Ataxia Telangiectasia Mutated; MX2: Myxovirus resistance 2; CCND1: Cyclin D1; NADSYN1: NAD synthetase 1; CYP2R1: Cytochrome P450, family 2, subfamily R, polypeptide 1; CASP8: Caspase 8; IRF4: Interferon regulatory factor 4; GWAS: Genome-Wide Association Study; SNP: Single Nucleotide Polymorphisms; OR: Odds Ratio; MAF: Minor Allele Frequency; PCR: Polymerase Chain Reaction; CI: Confidence Interval; HWE: Hardy-Weinberg Equilibrium; LD: Linkage Disequilibrium; TFBS ETS1: Transcription Factor Binding Site; ETS1: v-ets erythroblastosis virus E26 oncogene homolog 1; LSIL: Low grade Squamous Intraepithelial Lesion.

## Competing interests

The authors declare that they have no competing interests.

## Authors’ contribution

MPC, MBM, MIV, GR, CMC, MMG, CGF, BC, MM, JAA, and ALL have made substantial contributions to conception and design, or acquisition of data, or analysis and interpretation of data. MPC, MBM, MIV and GR, have been involved in drafting the manuscript or revising it critically for important intellectual content. All authors have given final approval of the version to be published.

## Pre-publication history

The pre-publication history for this paper can be accessed here:

http://www.biomedcentral.com/1471-2407/13/160/prepub

## Supplementary Material

Additional file 1 **Classification of the Spanish samples studied by age, sex and phenotype.** Categorization of the phenotypic characteristics studied in our population. P-values were obtained in order to test the differences between cases and controls. Click here for file

Additional file 2 **SNPs considered in our study and useful information.** Gene location, selected SNPs and their location, context sequence, and aminoacid changes. Click here for file

Additional file 3 **Genotypic association with phenotypic characteristics in the studied population.** Genotypic association between the SNPs selected for this study and the phenotypic traits statistically significant associated with melanoma. We have considered the most significant statistical model for the associated SNPs. For the SNPs that have no significant results we only show the genotypic model. Click here for file
