# Supplementary material for: Genetic variants in PARP1 (rs3219090) and IRF4 (rs12203592) genes associated with melanoma susceptibility in a Spanish population
Source: BMC Cancer. 2013 Mar 27;13:160. doi: 10.1186/1471-2407-13-160 (PMC3704782; doi:10.1186/1471-2407-13-160)
Supplement: Additional file 1 — Classification of the Spanish samples studied by age, sex and phenotype. Categorization of the phenotypic characteristics studied in our population. P-values were obtained in order to test the differences between cases and controls. [file 1471-2407-13-160-S1.docx]

| Characteristic | Controls  (N=347) | Cases  (N=566) | P-Fisher | OR (95%) | p-value |
| --- | --- | --- | --- | --- | --- |
| SEX  Male  Female  Unknown | 148 (42.65)  172 (49.57)  27 (7.78) | 254 (44.88)  305 (53.89)  7 (1.24) | 0.83 | 0.97 (0.74-1.28) | 0.82 |
| AGE AT DIAGNOSIS  <Mean  >Mean  Mean(SD)  Median  Unknown | 127 (36.6)  103 (29.68)  53.3 (15.81)  52  117 (33.72) | 259 (45.76)  273 (48.23)  52.83 (15.73)  54  34 (6.01) | 0.11 | 1.3 (0.95-1.77) | 0.1 |
| SOLAR EXPOSURE  No  Yes  Unknown | 25 (7.20)  231 (66.57)  91 (26.22) | 44 (7.77)  512 (90.46)  10 (1.77) | 0.42 | 1.26 (0.75-2.11) | 0.38 |
| CHILDHOOD SUNBURN  No  Yes  Unknown | 216 (62.25)  71 (20.46)  60 (17.29) | 161 (28.45)  364 (64.31)  41 (7.24) | **1.97*10⁻¹⁴** | 6.88 (4.96-9.53) | **4.52*10⁻³¹** |
| NEVI  <50  >50  Unknown | 257 (74.06)  30 (8.65)  60 (17.29) | 472 (83.39)  85 (15.02)  9 (1.59) | 0.06 | 1.54 (0.99-2.40) | 0.06 |
| LENTIGINES  No  Yes  Unknown | 163 (46.97)  129 (37.18)  55 (15.85) | 161 (28.45)  396 (69.96)  9 (1.59) | **6.63*10⁻¹⁴** | 3.11 (2.31-4.17) | **4.73*10⁻¹⁴** |
| HAIR COLOUR  Brown/Black  Blond/Red  Unknown | 296 (85.3)  25 (7.2)  26 (7.49) | 425 (75.09)  128 (22.61)  13 (2.3) | **2.11*10⁻⁹** | 3.57 (2.27-5.61) | **3.92*10⁻⁸** |
| EYE COLOUR  Dark  Light  Unknown | 247 (71.18)  69 (19.88)  31 (8.93) | 331 (58.48)  226 (39.93)  9 (1.59) | **1.31*10¯⁸** | 2.44 (1.78-3.52) | **2.96*10⁻⁸** |
| SKIN COLOUR  Dark  Light  Unknown | 139 (40.06)  176 (50.72)  32 (9.22) | 227 (40.11)  321 (56.71)  18 (3.18) | 0.47 | 1.12 (0.84-1.48) | 0.44 |

Additional File 1. Classification of the Spanish samples studied by age, sex and phenotype.

Bold denotes statistically significant p-values.
