# Supplementary material for: Genetic variants in PARP1 (rs3219090) and IRF4 (rs12203592) genes associated with melanoma susceptibility in a Spanish population
Source: BMC Cancer. 2013 Mar 27;13:160. doi: 10.1186/1471-2407-13-160 (PMC3704782; doi:10.1186/1471-2407-13-160)
Supplement: Additional file 2 — SNPs considered in our study and useful information. Gene location, selected SNPs and their location, context sequence, and aminoacid changes. [file 1471-2407-13-160-S2.docx]

Additional File 2. SNPs considered in our study and useful information.

| **GENE** | **MIM code** | **GENE LOCATION** | **SNP** | **SNP LOCATION** | **Protein encoded** | **aa change** | **Nt change** | **MAF (CEU)** | **Sequence context** |
| --- | --- | --- | --- | --- | --- | --- | --- | --- | --- |
| *PARP1* | 173870 | 1q41-q42 | rs3219090 | Intron 13 | Chromatin-associated enzyme, poly(ADP-ribosyl)transferase |  | G>A | 0.363 | ATAGTAAACATACTACCAAATGTTGA[A/G]AGGAGAATAATAGTCCTTCACAAAT |
| *CASP8* | 601763 | 2q33-q34 | rs13016963 | Intron 12 | Caspase |  | G>A | 0.416 | CAAGGTTAGAGGAAGGAAACATCAGC[A/G]AGGAGCAGGTGGAACAATCCCCAGG |
| *MX2* | 147890 | 21q22.3 | rs45430 | Intron 1 | Protein of dynamin and GTPases families |  | A>G | 0.327 | TGGGGCTGACCGTTCCTCCTGAGGAT[A/G]TGGGAGGGTTCCGTACTATTACTTA |
| *CYP2R1* | 608713 | 11p15.2 | rs10741657 | 5’UPST | Cytochrome P450 |  | G>A | 0.373 | GTGGTTGGGGAGATACTTTAGCAGGC[A/G]AGGGCTGTCAAGGAAAGTCTTATTA |
| *CCND1* | 168461 | 11q13 | rs1485993 | 5'UPST | Cyclin |  | C>T | 0.357 | GCTGGAGCTTTTCTTTGCTCCAAAGT[C/T]GACTTTGTCTGAAATTAATGTAGCT |
| *NADSYN1* | 608285 | 11q13.4 | rs7944926 | Intron 1 | Nicotinamide adenine dinucleotide synthetase |  | G>A | 0.277 | TCATTTGCTCAGAGCAAATCTAGTTG[A/G]ACTGAAGAAGGCTTGGCCAAAACTA |
|  |  |  | rs12785878 | Intron 2 |  |  | T>G | 0.274 | TGGGCTGTCTGATATCACAAAGCTTC[G/T]ATCCTCTCCTGGCCCCGTGGCCGGA |
| *ATM* | 607585 | 11q22-q23 | rs1801516 | Exon 34 | PI3/PI4-kinase | D1853N | G>A | 0.188 | ACTTGATTCATGATATTTTACTCCAA[A/G]ATACAAATGAATCATGGAGAAATCT |
| *IRF4* | 601900 | 6p25-p23 | rs12203592 | Intron 4 | Interferon regulatory factor family of transcription factors, |  | C>T | 0.159 | CATCCACTTTGGTGGGTAAAAGAAGG[C/T]AAATTCCCCTGTGGTACTTTTGGTG |

Location is described considering as the first Exon 1 of consensus sequence. DWST means downstream, UTR means untranscribed region and UPST means upstream.

Red in sequence context denotes nucleotide change.
