# Supplementary material for: Genetic variants in PARP1 (rs3219090) and IRF4 (rs12203592) genes associated with melanoma susceptibility in a Spanish population
Source: BMC Cancer. 2013 Mar 27;13:160. doi: 10.1186/1471-2407-13-160 (PMC3704782; doi:10.1186/1471-2407-13-160)
Supplement: Additional file 3 — Genotypic association with phenotypic characteristics in the studied population. Genotypic association between the SNPs selected for this study and the phenotypic traits statistically significant associated with melanoma. We have considered the most significant statistical model for the associated SNPs. For the SNPs that have no significant results we only show the genotypic model. [file 1471-2407-13-160-S3.docx]

Additional File 3. Genotypic association with phenotypic characteristics in the studied population.

| SNP(GENE) | EYE COLOR | | HAIR COLOR | | SKIN COLOR | | LENTIGINES | | NEVI | | CHILDHOOD SUNBURN | | BRESLOW INDEX | |
| --- | --- | --- | --- | --- | --- | --- | --- | --- | --- | --- | --- | --- | --- | --- |
|  | OR  (95% CI) | p-value | OR  (95% CI) | p-value | OR  (95% CI) | p-value | OR  (95% CI) | p-value | OR  (95% CI) | p-value | OR  (95% CI) | p-value | OR  (95% CI) | p-value |
| rs3219090 (*PARP1*) | **0.69 (0.54-0.88)** | **0.002** | 0.96 (0.72-1.28) | 0.803 | 0.85 (0.68-1.05) | 0.137 | 0.94 (0.75-1.18) | 0.599 | 0.92 (0.67-1.27) | 0.621 | 0.97 (0.78-1.22) | 0.824 | 1.04 (0.74-1.48) | 0.806 |
| rs13016963 (*CASP8*) | 0.83 (0.67- 1.03) | 0.1 | 0.94 (0.72- 1.23) | 0.645 | 0.96 (0.78- 1.18) | 0.705 | 1.1 (0.88- 1.34) | 0.438 | 0.81 (0.6- 1.1) | 0.178 | 1.11 (0.9- 1.36) | 0.346 | 0.94 (0.68- 1.29) | 0.697 |
| rs45430 (M*X2*) | 1.03 (0.83- 1.28) | 0.764 | 1.08 (0.83- 1.42) | 0.547 | 1.11 (0.9- 1.36) | 0.342 | 1.08 (0.88- 1.34) | 0.455 | 1.25 (0.94- 1.68) | 0.130 | 1.13 (0.91- 1.4) | 0.254 | 1.2 (0.87- 1.65) | 0.256 |
| rs10741657 (*CYP2R1*) | 1.10 (0.88- 1.38) | 0.392 | 1.11 (0.84- 1.47) | 0.448 | 1.24 (1- 1.54) | **0.045** | 0.84 (0.68- 1.05) | 0.125 | 0.82 (0.6- 1.13) | 0.226 | 0.97 (0.78- 1.21) | 0.814 | 1.33 (0.95- 1.86) | 0.098 |
| rs1485993 (*CCND1*) | 0.56 (0.41- 0.78) | **4.96*10⁻⁴** | 0.85 (0.64- 1.12) | 0.256 | 0.81 (0.66- 1) | 0.053 | 0.92 (0.74- 1.14) | 0.446 | 1.03 (0.76- 1.39) | 0.850 | 0.96 (0.78- 1.19) | 0.713 | 0.49 (0.25- 0.96) | **0.039** |
| rs7944926 (*NADSYN1*) | 1.08 (0.86- 1.34) | 0.509 | 1.15 (0.88-1.51) | 0.307 | 1.37 (1.03- 1.84) | **0.033** | 0.83 (0.67- 1.03) | 0.092 | 1.59 (1.01- 2.48) | **0.044** | 0.85 (0.68-1.05) | 0.139 | 1.26 (0.89- 1.78) | 0.186 |
| rs12785878 (*NADSYN1*) | 1.06 (0.85- 1.32) | 0.608 | 1.14 (0.86- 1.51) | 0.360 | 1.15 (0.93- 1.43) | 0.189 | 0.82 (0.66- 1.02) | 0.078 | 1.24 (0.92- 1.67) | 0.159 | 0.69 (0.51- 0.93) | **0.015** | 1.35 (0.95- 1.92) | 0.095 |
| rs1801516 (*ATM*) | 0.99 (0.72-1.37) | 0.968 | 0.73 (0.47-1.13) | 0.160 | 0.93 (0.69-1.25) | 0.635 | 1.04 (0.76-1.42) | 0.801 | 3.12 (1.06-9.2) | **0.039** | 0.85 (0.62-1.16) | 0.307 | 0.9 (0.54-1.52) | 0.7 |
| rs12203592 (*IRF4*) | 1.83 (1.34-2.51) | **1.63*10⁻⁴** | 0.18 (0.84-1.65) | 0.346 | 1.32 (0.99-1.74) | 0.054 | 1.61 (1.16-2.24) | **0.005** | 0.91 (0.6-1.37) | 0.641 | 1.06 (0.81-1.41) | 0.655 | 0.91 (0.6-1.4) | 0.679 |

OR means Odds Ratio. CI means Confidence Interval.

Bold denotes statistically significant p-values and their Odds Ratio according to the most significant model (genotipyc, codominant or recessive).
